# EphrinA4 plays a critical role in α4 and αL mediated survival of human CLL cells during extravasation

**DOI:** 10.18632/oncotarget.10311

**Published:** 2016-06-27

**Authors:** Miguel A. Flores, Paula Fortea, Eva M. Trinidad, Dolores García, Gloria Soler, Francisco J. Ortuño, Agustín G. Zapata, Luis M. Alonso

**Affiliations:** ^1^ Cytometry and Fluorescence Microscopy Research Center, Universidad Complutense de Madrid, 28040 Madrid, Spain; ^2^ Hematology and Medical Oncology Department, HGU Morales Meseguer, Marqués de los Velez, 30008 Murcia, Spain; ^3^ Department of Cell Biology, Faculty of Biology, Universidad Complutense de Madrid, José Antonio Nováis, 2, 28040 Madrid, Spain; ^4^ Transformation and Metastasis Group, Cancer Epigenetic and Molecular Biology Program (PEBC), IDIBELL, 08908 Barcelona, Spain

**Keywords:** leukemia, extravasation, apoptosis, integrin, ephrin

## Abstract

A role of endothelial cells in the survival of CLL cells during extravasation is presently unknown. Herein we show that CLL cells but not normal B cells can receive apoptotic signals through physical contact with TNF-α activated endothelium impairing survival in transendothelial migration (TEM) assays. In addition, the CLL cells of patients having lymphadenopathy (LApos) show a survival advantage during TEM that can be linked to increased expression of α4 and αL integrin chains. Within this context, ephrinA4 expressed on the surface of CLL cells sequestrates integrins and inactivates them resulting in reduced adhesion and inhibition of apoptotic/survival signals through them. In agreement, ephrinA4 silencing resulted in increased survival of CLL cells of LApos patients but not LA neg patients. Similarly was observed when a soluble ephrinA4 isoform was added to TEM assays strongly suggesting that accumulation of this isoform in the serum of LApos patients could contribute to CLL cells dissemination and survival in vivo. In supporting, CLL lymphadenopathies showed a preferential accumulation of apoptotic CLL cells around high endothelial venules lacking ephrinA4. Moreover, soluble ephrinA4 isolated from sera of patients increased the number and viability of CLL cells recovered from the lymph nodes of adoptively transferred mice. Finally, we present evidence suggesting that soluble ephrinA4 mediated survival during TEM could enhance a transcellular TEM route of the CLL cells. Together these findings point to an important role of ephrinA4 in the nodal dissemination of CLL cells governing extravasation and survival.

## INTRODUCTION

Compelling evidence supports that CLL cell survival within the tumor microenvironment is highly dependent on extrinsic signals provided by non-leukemic cell types [1, 2]. Endothelial cells can protect CLL cells from spontaneous apoptosis in vitro through soluble factors and/or direct physical contacts [1–7]. Both findings suggest that endothelial cells could play a similar role during extravasation an issue that, to our knowledge, has not been previously addressed. It has been emphasized a major effect of transendothelial migration (TEM) in the survival of non-leukemia cell types including granulocytes [8], T lymphocytes [9] or CD34+CD14+ monocyte precursors [10]. In agreement, the extravasation through inflamed vascular vessels or the specialized high endothelial venules (HEV) in lymphoid tissues [11] could affect the survival outcome of CLL cells.

In CLL cells, the TEM capacity is strongly dependent on α4 (CD49d) expression which together with αL integrin (CD11a) contributes to enhance it in patients having lymphadenopathy [12–14]. CD49d can also mediate contact dependent survival of CLL cells within tumor microenvironment [6, 15] associating with inferior prognosis groups including unmutated (UM) IgHV or CD38 expressing cases. Together these lines of evidence strongly suggest that integrin dependent extravasation may be further linked to a survival advantage that needs, however, a definitive demonstration.

Moreover, CLL cells show a reduced TEM compared to that of normal B cells as demonstrated under static or flow in vitro TEM conditions [12, 14, 16] and in an adoptively transferred mice in vivo model [12]. We showed that this can be in part linked to overexpression of ephrinA4, a GPI-membrane-linked ligand of the Eph receptor family of tyrosine kinases which is found as a membrane bound and a soluble isoforms in CLL and normal B cells [17, 18]. Reverse signaling through the cell surface expressed isoform inhibits integrin mediated adhesions of CLL cells to endothelium through binding to EphA2 receptor on the surface of endothelial cells [16, 19] likely impairing extravasation. By contrast, the soluble isoform enhances TEM of CLL cells in vitro when bound by endothelial cells through EphA2. This process could be coupled to enhanced diapedesis through sequestration and internalization of ICAM-1 and VCAM-1 [16] rather than through increased vascular permeability of the altered endothelial junctions found after ephrinA1 treatment [20]. Moreover patients having lymphadenopathy show increased serum levels of ephrinA4 in addition to decreased expression of ephrinA4 on the surface of CLL cells [18]. Together these findings pointed to soluble isoform as a likely mechanism contributing to nodal dissemination of CLL cells which needed further confirmation in a more physiological in vivo setting [21–23]. Herein we present for the first time in vitro and in vivo evidence suggesting that the major role of two ephrin A4 isoforms in CLL could be related with a non-previously described mechanism of survival linked to extravasation strongly dependent on integrin signaling.

## RESULTS

### CLL cells, but not normal B cells, can suffer apoptosis through physical contact with TNF-α activated endothelium resulting in impaired survival after transendothelial migration (TEM)

**Figure 1:**
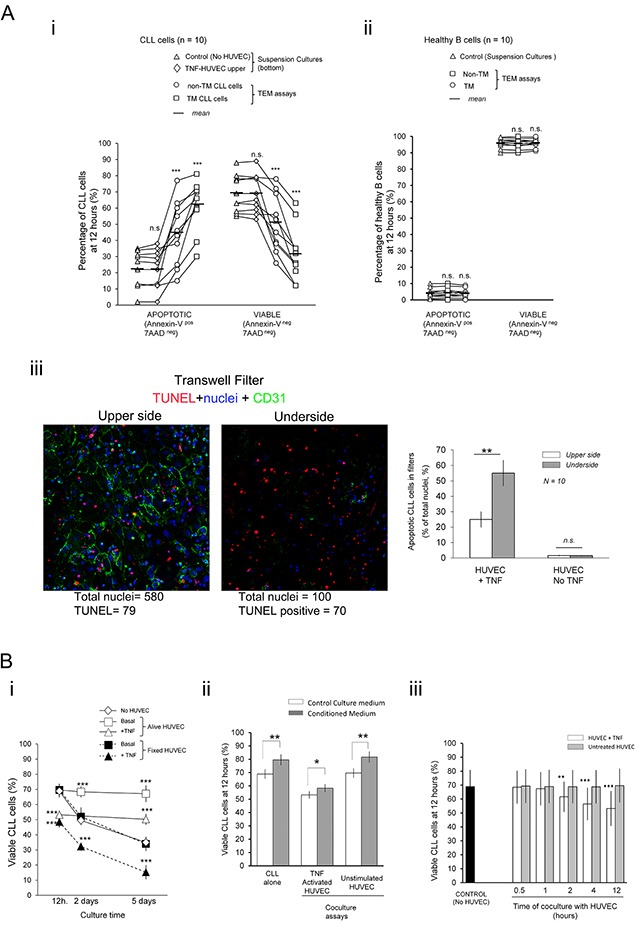
Physical contacts of CLL cells with TNF-αactivated endothelial cell monolayers during transendothelial migration (TEM) impair survival of leukemia cells **A.** CLL cells from 10 patients (n. 1-10; Table 1) (i) or normal B cells isolated from the peripheral blood of 10 healthy donors (ii) were added onto TNFα pretreated confluent monolayer of HUVEC (TNF-HUVEC) in transwell plates (Transendothelial Migration (TEM) assays) or to the bottom compartment of separate transwells (Suspension cultures)(5×10^5^ cells/well). Suspension cultures of CLL cells were carried out in bottom wells either with or without a TNF-HUVEC onto the upper filter. The percentage of apoptotic and viable cells in each experimental condition were determined after 12 hours by flow cytometry analysis of Annexin-V-PE/7AAD double staining. Each experimental condition was compared to basal levels in control suspension cultures without HUVEC (TEM assays: non-TM, non-transmigrated; TM, transmigrated). (iii) Transwell filters of TEM assays with CLL cells performed with TNF-α activated (+TNF) or untreated HUVEC (No TNF) were fixed and fluorescently stained with TUNEL (red; apoptotic nuclei), anti-CD31 (green; HUVEC junctions) and Hoechst (blue; total nuclei). Laser confocal microscopy images were taken from the upper and underside of filters (magnification 20x; ≥ 3 microscopy fields per filter; 1-2 filters per sample). Cartoons are from a representative TEM experiment with TNF-HUVEC. Total and TUNEL stained nuclei were counted (Image J; > 200 nuclei per sample and experimental condition) and the frequency of apoptotic cells in each side of the filters calculated (Right panel). Note that although the absolute number of nuclei was higher in the upper than the undersides the percentages of apoptotic CLL cells increased in the later one. Data are mean (±SD) from ten independent samples. **B.** i) CLL cells were cultured onto alive or paraformaldehyde fixed (fixed) TNF-α preactivated or untreated confluent HUVEC monolayers. The percentages of viable CLL cells (Annexin-V-neg 7AAD neg) were determined at the indicated time points by flow cytometry and compared to control suspension cultures without HUVEC. ii) CLL cells were cultured for 12 hours in suspension or onto alive HUVEC with normal culture medium or conditioned medium from 5 days CLL-HUVEC cocultures. iii) CLL cells were harvested at the indicated time-points from cocultures onto untreated or TNF-activated HUVEC and left in culture alone up to 12 hours. Two-tailed Student's t-test significance values: * P<0.05; ** P<0.01; *** P<0.001; n.s. non-significant.

The possible impact of extravasation in the survival of CLL cells was analyzed in the widely accepted [24] transendothelial migration (TEM) assays. HUVEC monolayers grown onto the filters were preactivated for 4 hour with TNF-α and then extensively washed in fresh culture medium before addition of the CLL cells onto them. The percentage of apoptotic (Annexin-V ^pos^ 7AAD ^neg^) and viable cells (Annexin-V ^neg^ 7AAD ^neg^) were measured by flow cytometry in the transmigrated (TM) and the non-transmigrated (non-TM) fractions after 4 or 12 hours. Values were compared to basal levels in control suspension cultures done in the bottom chambers of separate wells having or not a TNFα pretreated HUVEC monolayer onto the filter. A clear increase in the spontaneous apoptosis in control suspension cultures was only evidenced after 12 hours (Supplementary Figure S2) resulting in decreased viability irrespectively of the presence of a TNF-HUVEC monolayer on top of the filter (Figure 1A-i) discarding any effect of soluble factors from endothelium. The viability of CLL cells in the TEM assays was even more decreased in the two TEM fractions and, especially, in the TM cells compared to basal levels in the control suspension cultures in bottom chambers (Figure 1A-i) indicating that direct contact with endothelium was involved in these effects. Normal B cells suffered no significant changes in viability or an increased one in some samples (Figure 1A-ii) suggesting a proapoptotic effect of endothelium specifically in the CLL cells. Importantly, CLL cells transmigrating through non-activated HUVEC showed no significant changes in viability compared to basal levels (Supplementary Figure S3) highlighting that only activated endothelium mediated the apoptotic signals. The low cellular density in bottom chambers could explain the decreased viability of TM cells compared to the non-TM ones. However, no significant improvement in viability of TM cells was found when cell numbers in the bottom chambers were increased through direct addition of CLL cells at the initiation of the assays (Supplementary Figure S4). To further confirm that CLL cells could receive apoptotic signals while crossing the TNF-HUVEC monolayer we examined TUNEL stained filters through confocal microscopy. This demonstrated the occurrence of numerous apoptotic CLL cells in both sides of the filters with TNF-HUVEC compared to untreated HUVEC assays (Figure 1A-iii). Although absolute numbers of total and apoptotic CLL cells predominated in the upper side of filters (Figure 1A-iii, cartoons) the frequency of apoptotic cells was significantly higher in the underside (Figure 1A-iii) in agreement with a higher frequency of apoptotic cells in the bottom than the upper chambers, as determined by flow cytometry (Figure 1A-i). Together these data supported that CLL cells receive apoptotic signals during TEM.

Since previous studies had reported prosurvival effects of endothelium in CLL cells cocultured several days [1–7], we analyzed the endothelium role in CLL viability cocultured onto TNF-α or unstimulated HUVEC monolayers at 12 hours or at longer time points 2 or 5 days. In line with previous studies [1–7] we observed a prosurvival effect onto unstimulatedand to a lower extent TNF-HUVEC but not earlier than 2 days (Figure 1B-i). Indeed, these prosurvival effects with TNF-HUVEC were preceded by a drop in CLL viability at 12 hours (Figure 1B-i) in concert with our TEM assays. In addition, paraformaldehyde fixed instead of alive HUVEC monolayers lacked any prosurvival effects in the cocultured CLL cells and, more importantly, emphasized the proapoptotic ones onto TNF-HUVEC even at 2 or 5 days (Figure 1B-i) pointing to the release of prosurvival soluble factors in the alive conditions. Indeed, conditioned media from 5 days cocultures with alive HUVEC had prosurvival effects in the CLL cells cultured for 12 hours alone or under any coculture condition (Figure 1B-ii) definitively demonstrating that soluble prosurvival factors accumulate after prolonged coculture. We further concluded that 2 hours coculture of CLL cells with TNF-HUVEC was sufficient to receive proapoptotic signals that irreversibly led to their apoptosis when harvested and left in culture alone until 12 hours (Figure 1B-iii).

Together these data conclusively demonstrated that CLL cells suffer apoptotic signaling through direct contacts with TNF-HUVEC and likely during extravasation which needed further investigation.

### CLL cells of patients having lymphadenopathy (LApos) have a survival advantage during TEM mediated by α4 and αL integrin chains

**Figure 2:**
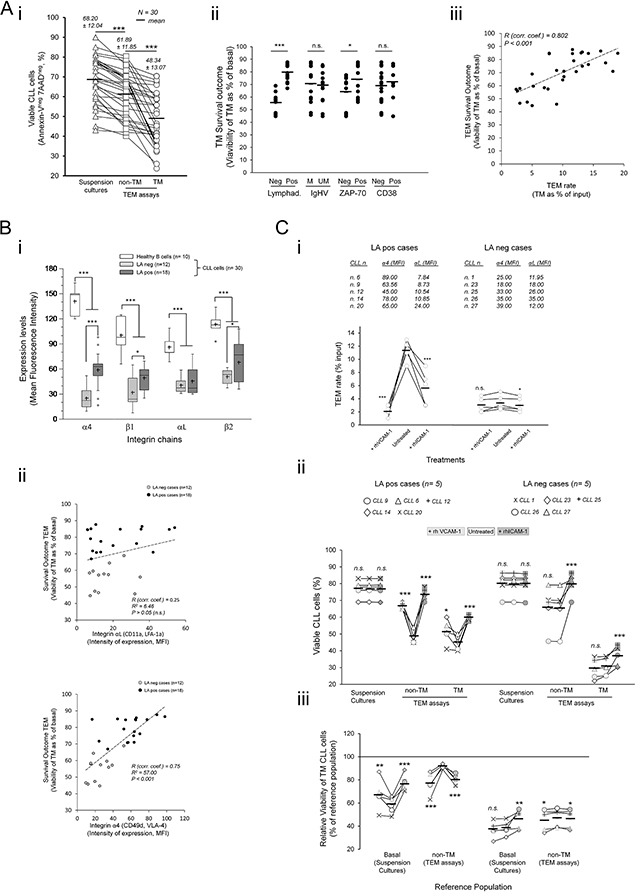
CLL cells of patients having lymphadenopathy (LApos) show a survival advantage during TEM mediated by αL and α4 integrins **A.** TEM assays through TNF-HUVEC were done with 30 patients and viability determined by flow cytometry analysis after 12 hours. i) Absolute values of viable cells. ii-iii) Viability of TM cells relative to basal levels in control suspension cultures without HUVEC (TM survival outcome) according to the indicated disease parameters (ii). iii) Linear correlation analysis between TM survival outcome and TEM rate. Absolute number of TM CLL cells, recovered from the bottom chambers, was measured by flow cytometry and expressed as percentage of total input cells (TEM rate). R, Spearman's correlation coefficient. **B.** Cell surface expression of the indicated integrin chains was measured in normal B cells and CLL cells by flow cytometry after staining with antigen specific antibodies. i) Whisper-box plots compare integrin expression levels (mean fluorescence intensity, MFI) between CLL cells and normal B cells, and between CLL cells of patients having or not lymphadenopathy (LApos and LAneg, respectively). ii) Linear correlation analysis between survival outcome of TM cells and expression of αL (upper plot) or α4 (lower plot) integrins. R, Spearman's correlation coefficient. **C.** CLL cells from 10 patients, having (LApos) or not (LAneg) lymphadenopathy, were preincubated or not (untreated) with recombinant soluble forms of human VCAM-1 (+rhVCAM-1) or ICAM-1 (+rhICAM-1) and left transmigrating through TNF-HUVEC monolayers (TEM assays) or cultured in suspension (suspension cultures) for 12 hours. Integrin expression levels (MFI) of the used samples are shown. i) TEM rate of samples were compared to that in control (untreated) TEM assays in LApos and LAneg cases. ii) Absolute levels of viable CLL cells in suspension cultures or in TEM assays (non-TM and TM cells) after rhVCAM-1 or rhICAM-1 preincubation. Treatments are compared to control (untreated) assays. iii) Viability of TM cells as percentage of a reference population: basal levels (suspension cultures) or non-TM cells. Paired two-tailed Student's T test significance values: * P<0.05; ** P<0.01; *** P<0.001.

Having stablished that TEM impairs survival of CLL cells, we examined its possible association with disease related parameters including lymphadenopathy (LA), IGHV gene mutational status or ZAP-70 or CD38 antigens expression. To this end, the sample size was increased by including 20 additional patients in TEM assays (Table 1). In all cases examined we confirmed a decline in the viability of both TEM fractions relative to basal levels and mainly in the TM cells (from 68% mean basal values to 48% mean values in the TM cells) (Figure 2A-i). Nevertheless, because the viability levels in TEM assays varied between samples we normalized them to the basal ones as a measure of survival outcome. This allowed us to find a significant association with the lymphadenopathy (LA) condition of patients and to a lower extent ZAP-70 rather than IGVH mutational status or CD38 expression (Figure 2A-ii). Samples having lymphadenopathy (LApos) showed a better survival outcome than those lacking it (LAneg) (Figure 2A-ii) suggesting a possible association with the TEM capacity. In supporting this conclusion we found a strong correlation of survival outcome with the TEM rate of samples (Figure 2A-iii) that largely associated with LA condition rather than other parameters (Supplementary Figure S5A).

**Table 1:** Clinical and molecular details of patients

N.	G.	Age(y)	Rai	LA	IgHV^a^	ZAP-70^b^	CD38^c^	del13q	del17p	del11q	+12	ephrinA4^d^	Integrin chain^e^			
													αL	β2	α4	β1
1	F	74	low	no	M	neg	neg	-	-	-	+	15.90	11.95	32.06	25.00	21.93
2	M	69	low	no	M	neg	pos	-	-	-	-	30.90	7.37	22.91	9.53	45.00
3	F	91	int	yes	UM	neg	pos	-	-	-	-	20.20	13.32	67.84	63.80	55.24
4	M	59	int	yes	M	neg	pos	-	-	-	-	7.70	13.20	57.45	24.28	50.74
5	M	78	hi	no	M	pos	neg	+	-	-	-	17.80	17.46	30.44	26.00	15.36
6	M	73	hi	yes	M	pos	pos	-	+	-	-	10.70	7.84	23.85	89.00	55.00
7	F	69	hi	yes	M	pos	neg	-	-	-	-	6.30	7.60	14.45	52.00	45.00
8	F	69	low	no	UM	neg	neg	+	-	-	-	36.47	9.92	13.69	19.60	7.58
9	F	78	int	yes	M	neg	neg	-	-	-	-	10.25	8.73	29.23	63.56	35.00
10	F	73	int	yes	M	neg	pos	+	-	-	-	7.83	42.12	65.56	56.78	59.23
11	F	65	hi	yes	UM	pos	neg	+	+	-	-	6.44	5.89	13.56	54.00	33.62
12	M	76	int	yes	UM	pos	neg	+	-	-	-	10.99	10.54	19.81	45.00	53.71
13	F	71	int	yes	M	pos	neg	-	-	-	+	12.62	35.48	56.68	16.53	69.67
14	F	58	hi	yes	M	neg	neg	-	-	-	-	10.46	10.85	21.07	78.00	63.00
15	M	69	int	yes	UM	pos	pos	+	-	-	-	9.63	51.00	54.00	56.00	25.00
16	F	63	int	yes	M	neg	neg.	+	-	-	+	11.25	20.00	23.00	98.00	65.00
17	M	79	int	no	UM	neg	neg	-	-	-	+	56.25	32.00	25.00	12.00	27.00
18	F	62	int	yes	M	neg	n.d.	-	-	-	-	6.98	8.00	68.00	34.00	32.00
19	F	85	int	yes	M	pos	neg	-	-	-	+	4.69	6.00	66.00	65.25	45.00
20	F	77	int	yes	UM	pos	neg	+	-	-	-	26.53	24.00	52.00	65.00	56.00
21	F	88	low	no	M	pos	neg	+	+	+	-	14.35	15.00	36.00	15.00	65.00
22	F	72	low	no	UM	pos	neg	+	-	+	-	62.25	7.00	35.00	15.00	53.00
23	M	75	low	no	UM	pos	neg	+	-	-	-	70.00	18.00	15.00	18.00	21.00
24	F	83	hi	yes	UM	neg	neg	-	-	-	-	18.69	33.00	84.00	70.00	45.00
25	F	58	low	no	M	pos	pos	-	+	-	-	68.00	26.00	29.00	33.00	56.00
26	F	66	low	no	UM	pos	neg	+	-	+	-	25.25	35.00	33.00	35.00	25.00
27	M	73	low	no	M	pos	neg	+	-	-	-	36.25	12.00	38.00	39.00	21.00
28	M	73	int	yes	M	pos	neg	-	-	-	-	14.56	54.00	61.00	66.00	68.00
29	M	68	low	no	UM	pos	pos	+	-	-	-	11.25	9.00	15.00	52.00	23.00
30	M	85	int	yes	UM	pos	pos	+	-	+	-	11.25	36.00	12.00	63.00	33.00

Footnotes:

a Sequences with a germline homology of ≥ 98% were considered as UM;

b, c Determined by flow cytometry (Positivity cut off over isotype stained negative control stainings): ZAP-70, > 25% stained cells; CD38, > 35% stained cells;

d, e Mean Fluorescence Intensity (MFI) determined by flow cytometry.

Because α4 integrin expression in LApos cases have been correlated with enhanced TEM capacity [12–14] and mediate survival signals from endothelium [6, 15], we investigated its possible association with TEM survival outcome. As determined by flow cytometry, the cell surface expression of α4 and αL integrins and their corresponding β chains were lower in CLL cells than in normal B cells although significantly increased in LApos respect to LAneg cases (Figure 2B-i) in concert with enhanced TEM rate (Supplementary Figure S5B-C). Interestingly, there was a strong correlation of α4, rather than αL expression, with the survival outcome of TM cells (Figure 2B-ii) further supporting a specific role of α4 integrin in this outcome.

To further confirm this, we blocked α4 or αL interactions in separate TEM assays through preincubating the CLL cells of LApos and, for comparison, LAneg cases with recombinant forms of VCAM-1 (rhVCAM-1) or ICAM-1 (rhICAM-1) endothelial ligands, respectively. Five samples having different α4 or αL expressions within each LA clinical conditions were compared (Figure 2C). LApos cases showed a strong reduction in the TEM rate after blocking either of the two integrin interactions (Figure 2C-i) in contrast to LAneg samples (Figure 2C-i) confirming that both integrin chains play a critical role in TEM of LApos cases. Moreover, the viability of both, non-TM and TM cells, was strongly improved in the LApos cases under blocking either of the two integrins compared to the untreated samples while only αL blocking did in the LAneg cases (Figure 2C-ii). The bound recombinant ligands had no significant effects in the viability of CLL cells in control suspension cultures (Figure 2C-ii) indicating that their effects in TEM assays can be linked to the inhibition of the corresponding integrin interactions with endothelium. The survival outcome of TM cells was greatly improved in the LApos cases compared to that in control untreated assays (Figure 2C-iii). However, it was impaired relative to the non-TM cells compared to the control cultures (Figure 2C-iii) suggesting that integrin interactions rescued the CLL cells during diapedesis from proapoptotic signals received at initial adhesions.

In conclusion, these data showed that the integrin adhesions responsible of the enhanced TEM of CLL cells are also critically involved in the survival outcome.

### Integrin dependent survival outcomes are counteracted by ephrinA4 reverse signaling

**Figure 3:**
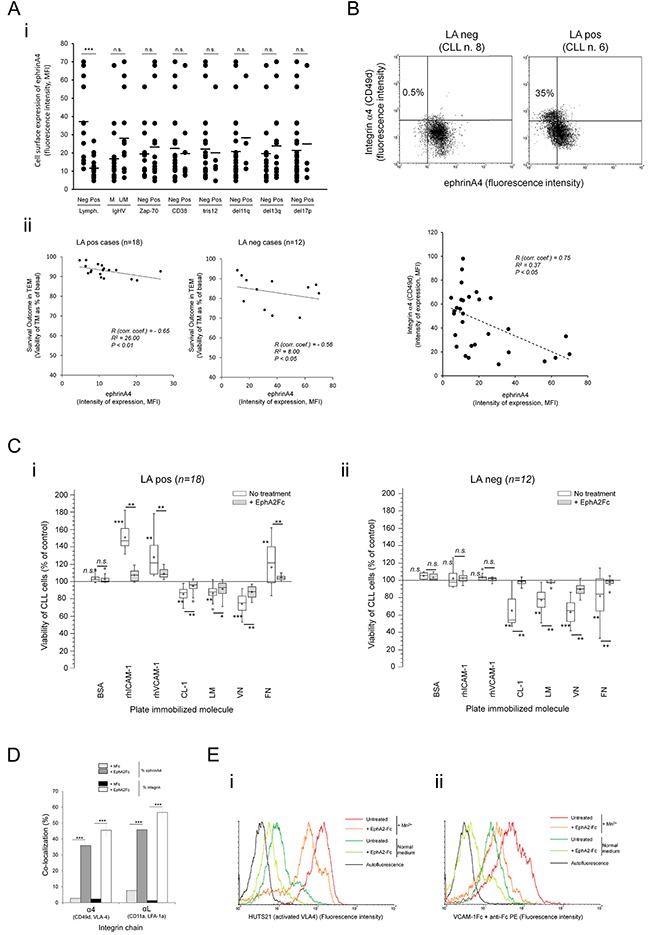
EphrinA4 reverse signaling suppresses integrin mediated survival signals through inhibiting activation state **A.** Expression of ephrinA4 was measured on the surface of CLL cells from 30 samples by flow cytometry analysis (MFI, mean fluorescence intensity) and (i) compared between samples according to disease parameters and cytogenetic characteristics or (ii) correlated with survival outcome of TM cells in LApos (left) and LAneg (right) cases. R: Spearman's correlation coefficient. **B.** Expression of ephrinA4 and CD49d (α4) on the surface of CLL cells was determined by flow cytometry (upper panels). Correlation analysis between ephrinA4 and α4 expression (mean fluorescence intensity, MFI) of samples. **C.** CLL cells from 30 patients (i, LApos; ii, LAneg) were preincubated (+EphA2Fc) or not (No treatment) with saturating amounts of soluble recombinant human EphA2 extracellular domains (EphA2Fc) and cultured for 12 hours in suspension in untreated plates (control) or plates containing the indicated molecules pre-immobilized to wells. BSA was used as a non-specific immobilized protein. Viability of CLL cells onto each protein is shown as percentage of basal levels in control suspension cultures without immobilized proteins. Statistical significances are shown for comparison between untreated cultures to control cultures or for EphA2Fc treatments to untreated conditions. Whisper-box plots: mean (+) and median (−) values. **D.** CLL cells from LApos samples (n=3) were preincubated in suspension with poly-His tagged EphA2Fc or hFc as control. Cell suspensions were then incubated with biotin anti-His followed by AlexaFluor488 streptavidin and adhered to slides for 15 min (37°C). After paraformaldehyde fixation (4% PF in PBS) slides were incubated with AlexaFluor647 conjugated monoclonal Ab for the indicated integrin chains and analyzed through confocal microscopy (Supplementary Figure S6). At least 3 fields per experiment were evaluated for colocalization analysis (Colocalization Tool, Image J). ≥ 200 cells per sample were analyzed. **E.** CLL cell suspensions (10^5^ /assay) were incubated for 30 min with EphA2Fc or Fc only in RPMI/2% FCS (Normal medium) or containing 1mM MnCl2 (Mn^2+^). Next, CLL cells were incubated with a PE coupled HUTS21 mAb in cold PBS (i) or PE coupled anti-Fc preclustered VCAM-1-Fc in normal medium (ii) followed by flow cytometry analysis. A representative LApos CLL sample is shown (n>3 samples). Paired two-tailed Student's T test significance values: * P<0.05; ** P<0.01; *** P<0.001.

Having stablished that integrins enhance the survival outcome of TM CLL cells in LApos patients, we analyzed its possible modulation through ephrinA4 reverse signaling as previously reported by us [16]. As determined by flow cytometry, ephrinA4 expression was significantly higher in LAneg than LApos samples (Figure 3A-i) while no significant differences were found according to ZAP-70, IGVH or CD38 parameters (Figure 3A-i) confirming its association with the LA condition of patients previously found by us [16, 18]. Remarkably, ephrinA4 expression levels were low in most trisomy 12 (tris12) cases (4 out of 5) and, to a lower extent, del11q positive samples (3 out of 4) (Figure 3A-i) which could be in concert with the increased risk to suffer lymphadenopathy in these patients. EphrinA4 expression and TEM survival followed an inverse correlation in both LA clinical groups (Figure 3A-ii) contrasting with that observed for integrins (Figure 2B-ii). Accordingly, expression of ephrinA4 and CD49d followed an opposite pattern and, in the LApos cases, the appearance of a CD49d high population correlated with a low ephrinA4 expression (Figure 3B). Together these data further supported a direct and/or indirect role of ephrinA4 in the survival events during TEM modulating integrin adhesions.

To discriminate between these two possibilities we preincubated the CLL cells from the 30 patients with saturating amounts of recombinant extracellular domains of human EphA2 (EphA2Fc) which results in ephrinA4 reverse signaling modulating integrin affinity [16]. The viability of CLL cells was analyzed, as in the preceding TEM assays, at 12 hours of culture in suspension alone or onto plate immobilized rhICAM-1 or rhVCAM-1 or other integrin ligands of the extracellular matrix (ECM) including fibronectin (FN), collagen type-1 (CL-1), laminin (LM) or vitronectin (VN). EphA2Fc did not significantly affect the viability of CLL cells cultured onto immobilized BSA relative to control assays (Figure 3C-i-ii) discarding a direct role of ephrinA4 reverse signaling in CLL cell viability. However, it largely counteracted most of the effects onto integrin ligands (Figure 3C-i-ii), including the prosurvival ones observed in LApos cases onto either of the two endothelial ligands. ECM molecules mainly induced a decrease in the viability of CLL cells of all samples (Figure 3C-i, ii) excluding FN which had prosurvival effects in LApos cases (Figure 3C-i-ii) confirming the critical role of α4 in this clinical group. These data agreed with prosurvival signals mediated by αL and, mainly, α4 integrins in LApos cases during diapedesis, as concluded from blocking them in the preceding TEM assays, and further highlighted a role of ephrinA4 in regulating them. In line with our previous findings [16], EphA2Fc induced a significant increase in the number of non-adhered CLL cells recovered from the adhesion assays (not shown) which occurred in the absence of a significant decrease in integrin expression (not shown). Confocal fluorescence microscopy demonstrated a lack of intracellular staining for integrins but rather showed their sequestration on the surface of the EphA2Fc treated CLL cells (Supplementary Figure S6) (Figure 3D). These results suggested that physical interactions on the cell surface rather internalization could modulate the integrin binding capacities. Indeed, flow cytometry analysis of HUTS-21 Abs staining, which recognizes high affinity conformations of VLA4 integrins, demonstrated that EphA2Fc treatment strongly inactivated VLA4 in the CLL cells even when artificially activated through addition of Mn^2++^ to medium (Figure 3E-i). Accordingly, binding of soluble VCAM-1-Fc by CLL cells was dramatically reduced in the presence of EphA2Fc (Figure 3E-ii). In conclusion, these data supported that ephrinA4 signaling inhibits integrin affinities for their ligands and, hence, apoptotic/survival signals.

### Absence of ephrinA4 reverse signaling dramatically improves the survival of TM CLL cells from LApos cases

**Figure 4:**
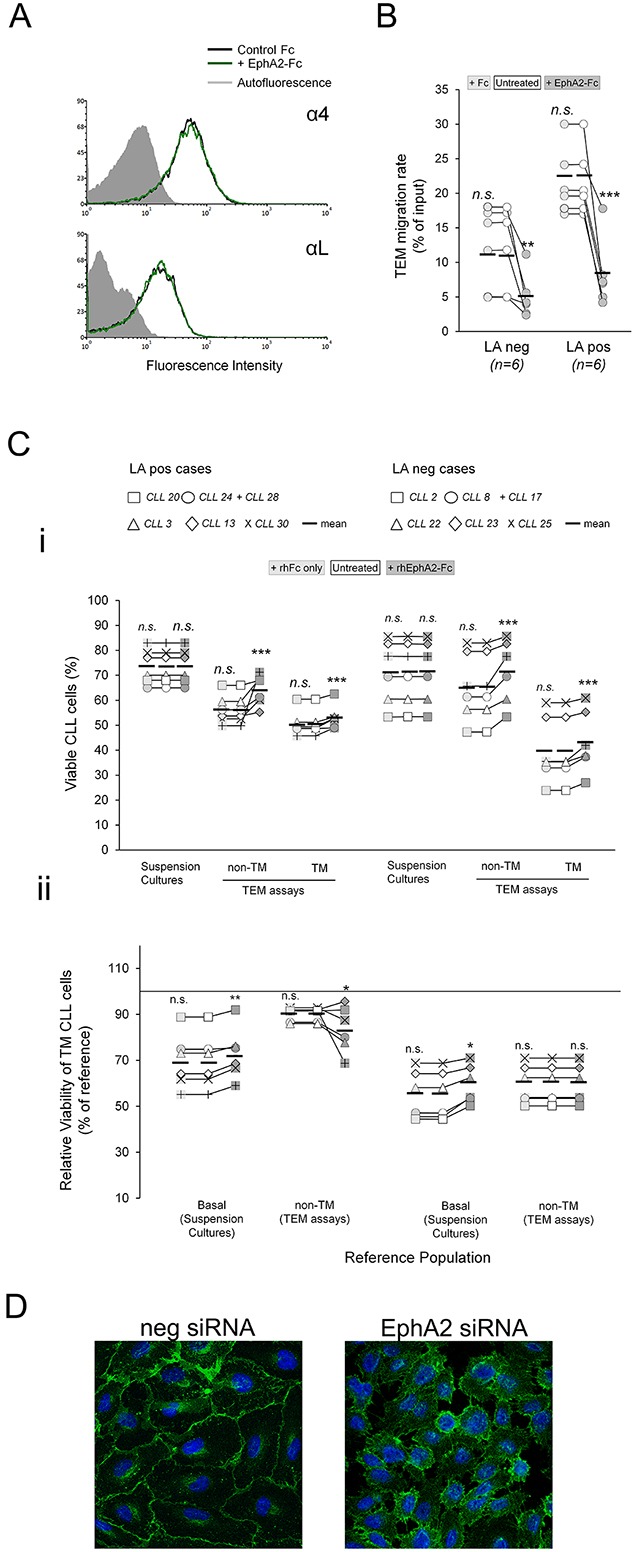
EphrinA4 reverse signaling inhibits survival signaling during diapedesis CLL cells from the indicated patients having (LApos) or not (LAneg) lymphadenopathy were preincubated with EphA2Fc or hFc or left untreated and allowed transmigrating through TNF-HUVEC monolayers (TEM assays) or cultured in suspension (suspension cultures) for 12 hours. **A.** Representative flow cytometry histograms of α4 or αL integrin expression in CLL cells before and after incubation with EphA2Fc. **B.** TEM rate of samples, as determined by flow cytometry, were compared between hFc or EphA2Fc treatments to untreated cultures in LApos and LAneg cases. **C.** Effects of EphA2Fc in viability of CLL cells. i) Absolute levels of viable CLL cells in suspension cultures or in TEM assays (non-TM and TM cells) after rhEphA2Fc or hFc treatment compared to control, untreated CLL cells. ii) Relative viability of TM cells as percentage of basal levels in suspension cultures or of the corresponding non-TM cells. **D.** HUVEC were nucleofected with EphA2 specific or negative control siRNA. VE-Cadherin (green) stained HUVEC cultures demonstrated that EphA2 silencing inhibited the formation of a proper confluent monolayer. Paired two-tailed Student's T test significance values: * P<0.05; ** P<0.01; *** P<0.001.

To demonstrate a role of ephrinA4 reverse signaling in TEM assays, CLL cells were preincubated with saturating amounts of the EphA2Fc recombinant molecules. Under these conditions, bidirectional signaling between CLL and endothelial cells through the receptor-ligand pair is inhibited while ephrinA4 reverse signaling into the CLL cells still can take place [16]. In these experiments, six samples of each LA condition were chosen that differed in ephrinA4 expression levels within each group (Figure 4C; Table 1). In line with our previous findings [16], rhEphA2 did not significantly changed integrin expression of CLL cells (Figure 4A) but led to a marked drop in the TEM rate of all samples (Figure 4B) in concert with the inhibition of integrin adhesions. Indeed, as in the integrin blocking TEM assays, it significantly increased both the viability of non-TM and TM cells in all samples (Figure 4C-i) and, more importantly, the survival outcome of TM cells relative to basal levels in suspension cultures but not relative to the non-TM ones of LApos cases (Figure 4C-ii). Altogether, these data supported that ephrinA4 reverse signaling during diapedesis likely inhibits integrin prosurvival signals into the CLL cells of LApos cases.

**Figure 5:**
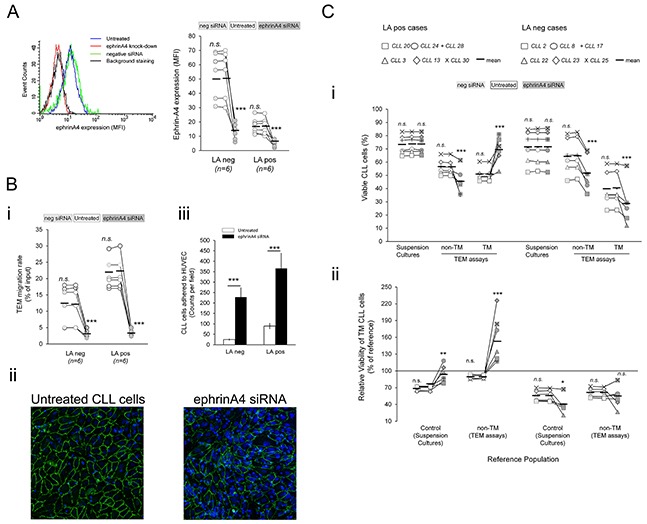
Absence of ephrinA4 reverse signaling during TEM increases survival outcome of TM cells in LApos samples CLL cells of the 12 patients used in EphA2Fc assays having (LApos) or not (LAneg) lymphadenopathy (Figure 4), were nucleofected with ephrinA4 specific or negative control siRNA duplexes or left untreated. **A.** EphrinA4 silencing was determined by flow cytometry 48 hours after nucleofection. **B.** Untreated or nucleofected cells (siRNA neg or ephrinA4) were left transmigrating for 12 hours through TNF-HUVEC. i) TEM rate of samples measured by flow cytometry. ii) Transwell filters from TEM assays were stained with anti CD31 mAb (green, HUVEC junctions) and Hoechst (blue, nuclei) and examined by confocal microscopy (40x oil immersion; Leica TCS-SP2). CD31 junctions were not altered in the HUVEC monolayer of ephrinA4 knock-down samples. iii) Absolute counts of CLL cells adhered to TNF-HUVEC were measured by image analysis (ImageJ; > 200 nuclei counted per filter). **C.** Analysis of CLL cells viability in TEM assays after ephrinA4 knock-down. i) Absolute levels of viable CLL cells in suspension cultures or in TEM assays (non-TM and TM cells) were compared between ephrinA4-specific or neg control siRNA nucleofected to control untreated CLL cells. ii) Relative viability of TM cells as percentage of basal levels in suspension cultures or of non-TM cells. Paired two-tailed Student's T test significance values: * P<0.05; ** P<0.01; *** P<0.001.

Next, we blocked ephrinA4 signaling in TEM assays through siRNA mediated silencing of EphA2 in the endothelial cells. However, absence of this receptor in the endothelial cells rendered them unable to form a tightly packed monolayer (Figure 4D) thus limiting further TEM assays. Next, we challenged ephrinA4 silencing in the CLL cells from the 6 LApos and LAneg cases used in the preceding EphA2Fc TEM. Forty-eight hours after nucleofection, we found a strong reduction of ephrinA4 expression on the cell surface (Figure 5A) without significantly compromising their viability as compared to untreated or control nucleofected samples. EphrinA4 knock-down had not significant effects on the expression of integrins but markedly reduced the TEM rate of samples as compared to siRNA neg or untreated CLL cells (Figure 5B-i) and the number of CLL cells retained onto the HUVEC monolayer increased as determined by fluorescent microscopy analysis of transwell filters (Figure 5Bii-iii). Collectively, these findings confirmed that ephrinA4 decreases adhesion [16, 18]. In terms of viability, ephrinA4 knock-down led to a marked decrease in the viability of non-TM cells of both LA clinical groups compared to siRNA neg nucleofected or untreated cells (Figure 5C-i) which was compatible with the increased adhesion and, hence, occurrence of proapoptotic signals at initial steps of TEM. By contrast, the viability of TM cells was dramatically improved in the LApos but not the LAneg cases compared to that in untreated samples and, more importantly, to their corresponding non-TM cells (Figure 5C-i) a finding also confirmed in terms of survival outcome (Figure 5C-ii). Together with the preceding integrin blocking or EphA2Fc TEM assays, these results supported that switching-off ephrinA4 reverse signaling during diapedesis represented a survival advantage for the CLL cells of LApos cases.

### Soluble ephrinA4 binding to endothelium strongly potentiates survival advantage of CLL LApos cases

**Figure 6:**
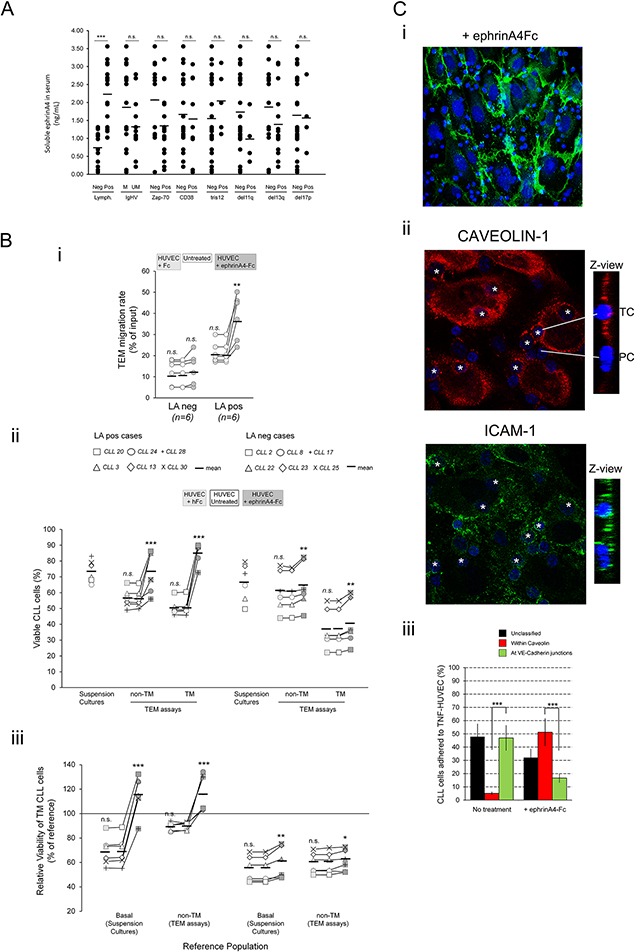
Treatment of TNF-HUVEC with soluble recombinant human ephrinA4 strongly potentiates survival of CLL cells and a possible transcellular TEM pathway **A.** Soluble ephrinA4 levels in the serum of CLL patients were determined by indirect ELISA (ng/mL) and possible associations with clinical parameters examined. **B.** CLL cells from 12 patients having (LApos) or not (LAneg) lymphadenopathy, used in the EphA2Fc or siRNA assays (Figures 3 and 4), were left transmigrating 12 hours through TNF-α preactivated HUVEC monolayers preincubated (30 min) with saturating amounts of recombinant human ephrinA4 (HUVEC+ephrinA4Fc), purified human Fc (HUVEC + hFc) or untreated before addition of CLL cells. i) Absolute number of transmigrated cells (TM) was counted by flow cytometry and expressed as percentage of total input cells (TEM rate). ii) Absolute levels of viable CLL cells in suspension cultures or in TEM assays (non-TM and TM cells) in the ephrinA4Fc or hFc treated TEM assays were compared to untreated conditions. iii) Relative viability of TM cells normalized to basal levels (suspension cultures) or to the corresponding non-TM cells in the ephrinA4Fc or hFc treatments compared to untreated TEM assays. **C.** i) Transwell filters were stained with anti VE-Cadherin (green, HUVEC junctions) and Hoechst (blue, nuclei) and examined by confocal microscopy. Adhered CLL cells were located at VE-Cadherin interendothelial junctions or separated from them. ii-iii) Caveolin-1 and ICAM-1 immunofluorescence staining of TEM assays done in chamber slides 2 hours after coculture with LApos CLL cells. ii) CLL cells inside of HUVEC were surrounded by caveolin-1 endothelial caveola (asterisks, > 1μm from VE-Cadherin junction) and ICAM-1. Insets show Z- views of caveolin and ICAM-1 staining for a a transcellular (TC) and a paracellular (PC) TEM CLL nuclei. iii) Measurement of CLL cells located within caveolin-1, at VE-Cadherin junctions or unclassified in ephrinA4Fc treated compared to untreated TNF-HUVEC after 2 hours coculture (at least 200 nuclei were counted per condition). Paired two-tailed Student's T test significance values: * P<0.05; ** P<0.01; *** P<0.001.

As previously demonstrated by us [18], herein we confirmed higher levels of soluble ephrinA4 in the serum from patients having lymphadenopathy than in those lacking it (Figure 6A) whereas a clear association with other parameters was not found (Figure 6A). Mechanistically, soluble ephrinA4 enhances extravasation of CLL cells after binding to EphA2 receptor on the surface of endothelial cells [16] through inhibiting cell surface expressed ephrinA4 mediated reverse signaling, thus allowing integrin mediated adhesions. Furthermore, EphA2 mediated sequestration and internalization of VCAM-1 and ICAM-1 endothelial ligands could be involved in this process [18]. To analyze whether this mechanism could contribute to improve the survival of CLL cells in TEM assays, we preincubated the TNF-HUVEC monolayer with saturating amounts of soluble recombinant human ephrinA4 (ephrinA4Fc) as previously described [18]. This resulted in an increased TEM rate of LApos but not the LAneg cases (Figure 6B-i) confirming our previous findings [16]. Moreover, the viability of non-TM and TM cells of LApos samples was dramatically improved (Figure 6B-ii) along with an enhancement in the survival outcome of TM cells compared to untreated control TEM assays (Figure 6B-iii), confirming a prosurvival role of soluble ephrinA4 during TEM of LApos CLL cells.

Next, immunofluorescence microscopy analysis of the transwell filters showed that VE-Cadherin^+^ junctions between HUVECs were unaltered in the ephrinA4Fc treatments (Figure 6C-i) discarding that CLL cells traversed through holes as a possible explanation to the increased survival. Moreover, CLL cells located at VE-Cadherin junctions (Figure 6C-i), suggesting a paracellular (PC) TEM route, or at > 1μm from them possibly following a transcellular (TC) TEM routes, as previously demonstrated [25]. To confirm a TC TEM pathway, we examined by confocal microscopy a possible association between intracellular caveolin-1 staining, which has been related with this TEM route in lymphocytes [26, 27], and the CLL cells actually inside of endothelial cells located > 1μm of interendothelial junctions. Many CLL cells inside of HUVEC were distinctly located within caveolin-1^+^ endothelial vacuoles and surrounded by ICAM-1 staining (Figure 6C-ii) suggesting a TC route of TEM. Image based counting of CLL cells at VE-Cadherin junctions or > 1μm within caveolin-1 in TEM assays revealed an increased frequency of the later under ephrinA4Fc treatments (Figure 6C-iii) in concert with an enhanced TC pathway through the endothelial cell body. These data confirmed that soluble ephrinA4Fc does not induce a TEM of CLL cells through disassembling interendothelial junctions but rather through endothelial cells themselves likely through a TC route of TEM.

### EphrinA4 can be detected in the HEV of CLL lymphadenopathies and enhances viability of CLL cells infiltrating the lymph node of adoptively transferred mice

**Figure 7:**
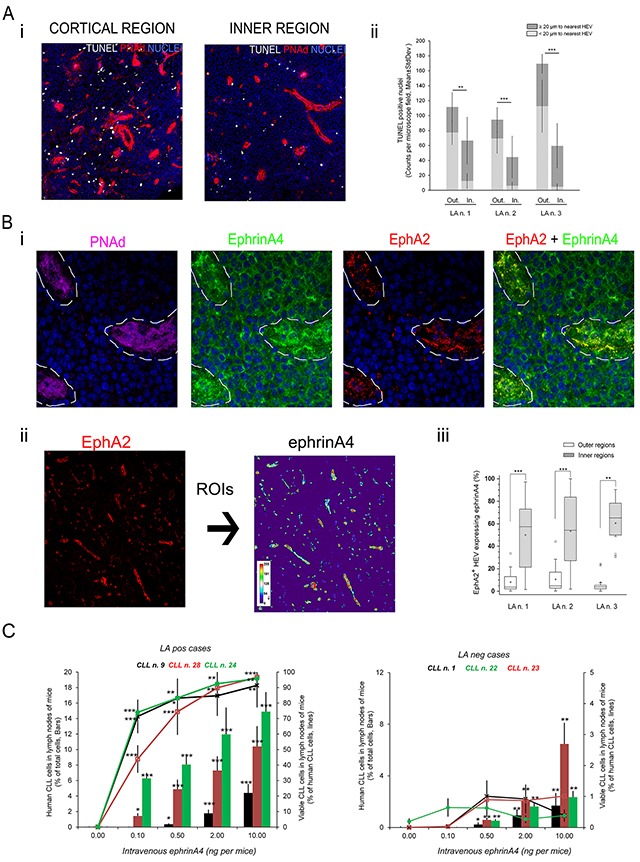
Soluble human ephrinA4 in HEV of lymphadenopathies inversely correlates with apoptotic cells near HEV dramatically enhancing the number and viability of human CLL cells recovered from lymph node of adoptively transferred mice **A-B.** Seven μm thick sections from frozen CLL lymphadenopathies (LA n.1, 2 and 3 corresponding to CLL patients n. 3, 18 and 28, respectively) were fixed in acetone and sequentially incubated with A) TUNEL (white) and antibodies against PNAd (red) or B) antibodies against PNAd (magenta), ephrinA4 (green), EphA2 (red). In all cases nuclei were counterstained with Hoechst (blue). Fluorescent images were taken in a laser confocal microscope (TCS SP-2 AOBS, Leica). A) Representative low magnification images (20x) of areas near the capsule (“Cortical” region, left panel) or deeper (>200um far from capsule, “Inner” regions; right panel). ii) Frequency of apoptotic nuclei around (≤ 20 μm) or separated from HEV in cortical and inner LA regions (*p<0.05; **p<0.01; ***p<0.001). B) i) HEV (PNAd+, magenta; dotted line) showed strong ephrinA4 staining co-localizing with EphA2. (63x). ii) Regions of interest (ROI) were traced around HEV according to EphA2 staining (left). Intensity of ephrinA4 staining within ROIs was measured (pseudocolor scale inset). iii) HEVs containing ≥ 10% pixels above 200 gray-scales were considered as positive. Whisper-box plots of > 20 measured fields from each LA section. Unpaired two-tailed Student's t-test significance values were *p<0.01; **p<0.005; ***p<0.001. **C.** Human CLL cells were stained with CFSE fluorescent tracer and adoptively transferred into germ-free young Balb/c mice (2×10^7^ per mice) by intravenous injection through the tail vein along with increasing concentrations of human soluble ephrinA4 purified from the sera of patients. Mice were sacrificed 24 hours later and cell suspensions from popliteal lymph nodes stained with an anti-mouse CD45 antibody, AnnexinV-PE and 7AAD for flow cytometry analysis. Human CLL cells were gated according to CFSE positivity and negativity for anti-mouse CD45 (Suppl. Figure S1). Data (mean±SD) were compared to mice receiving human CLL cells in PBS without ephrinA4. Five animals were used per sample and experimental condition. Paired two-tailed Student's T test significance values were *p<0.05; **p<0.01; ***p<0.001.

A possible correlation between apoptosis and in vivo extravasation was indirectly investigated by immunofluorescence microscopy analysis of CLL lymphadenopathies. Apoptotic cells were detected in tissue sections through TUNEL staining and their location relative to HEV through PNAd staining. At low magnification, we confirmed an accumulation of apoptotic cells within 200 μm areas under the capsule in the three LA studied (Figure 7A-i). Because normal lymph node architecture is profoundly effaced in CLL lymphadenopathies we referred to these areas concentrating apoptotic cells near the capsule as outer rather than cortical regions, and as inner regions to the deeper ones inside of the LA parenchyma. Interestingly, apoptotic cells were closer to HEV in outer than inner regions (Figure 7A-i) indirectly suggesting an association with extravasation. After extravasation normal lymphocytes are retained for hours within an area of < 20 μm around the HEV of cortical lymph node regions before infiltrating the LN parenchyma [28], a feature that correlated with an accumulation of apoptotic cells around them. Indeed, we found that the number of apoptotic nuclei within ≤ 20 μm around the HEV was markedly higher in the outer than the inner LA areas (Figure 7A-ii) suggesting that they could represent recently extravasated CLL cells. Its possible association with ephrinA4 staining was further examined. EphrinA4 staining was found not only in the infiltrated CLL cells but also within the endothelial cells of HEV bound to EphA2 (Figure 7B-i). Quantification of ephrinA4 staining bound to EphA2 in HEV in outer and inner regions (Figure 7B-ii) revealed a significant higher frequency in the former (Figure 7B-iii) thus following a completely opposed distribution to that observed for apoptotic cells. Altogether these data supported a possible in vivo prosurvival role of soluble ephrinA4 related to the extravasation of CLL cells into lymphadenopathies.

To assess this in a more physiological condition, CFSE stained human CLL cells of LApos and LAneg patients were adoptively transferred to Balb/c mice receiving different amounts of a soluble ephrinA4 purified from the sera of CLL patients. The percentage and viability of CLL cells was analyzed 24 hours later by flow cytometry (Supplementary Figure S1) in cell suspensions of the popliteal lymph node where the number of endogenous mouse lymphocytes is greatly lower than in other lymph nodes. EphrinA4 led to an increase in the proportion (Figure 7C, Bars) and viability (Figure 7C, lines) of the human CLL cells recovered from the lymph nodes of mice inoculated with LApos samples in a concentration-dependent manner.

## DISCUSSION

Herein we provide evidence, for the first time, that CLL cells establish physical contacts with endothelia that can result in decreased survival during extravasation. In patients having lymphadenopathy, CLL cells are able of collecting prosurvival signals through αLβ2 and α4β1 integrins linked to ICAM-1 and VCAM-1 mediated diapedesis. In these processes, the soluble form of ephrinA4, that accumulates in the sera of patients having lymphadenopathy [18], plays an instrumental role. In these patients, ephrinA4 binding to EphA2 receptor on the luminal side of endothelial cells facilitates the adherence and migration of CLL cells through the endothelial vessels of lymphoid tissues, coupling enhanced TEM to prosurvival signals. Thus, the present findings expand our previous studies on the role of ephrinA4 isoforms in CLL cell adhesion and TEM [16, 18] by further connecting them to CLL cell survival and lymphadenopathy.

Our present results shed new light on the role of endothelial cells in CLL. In accordance with previous studies [3, 4, 6, 29–31] we demonstrate that endothelial cells can provide prosurvival signals to the leukemia cells within infiltrated tissues which are largely mediated by the release of prosurvival soluble factors. This is dependent on physical contacts between both cell types likely favored by prolonged coexistence within the infiltrated tissues. In supporting this hypothesis we demonstrated a lack of prosurvival effects in prolonged cocultures when using PF fixed rather than alive HUVEC. Besides, conditioned medium from long term cocultures protected the CLL cells from spontaneous and TNF-HUVEC mediated apoptosis in overnight (12 hours) cultures. By contrast, the timeframe of extravasation process is not presumably long enough [27] to promote the release of soluble factors by endothelial cells while proapoptotic signals through physical contacts could predominate. This is supported by the increase of apoptotic CLL cells in the non-TM cells and can likely take place as early as 2 hours after contact as further demonstrated in cocultures. It would be interesting to confirm our results under flow conditions in a model recently used by other authors [13, 32] or through adapting flow conditions to transwells [33]. During diapedesis the CLL cells can be rescued from propapoptotic signals received at initiation of TEM in LApos cases. However, we cannot exclude that the migrated CLL cells could receive other survival signals once inside the lymph node parenchyma although our studies in LN sections show numerous apoptotic cells near to HEV. These data suggest that many recently migrated cells die without infiltrating the tissue and especially when migrating through the HEV lacking ephrinA4 that predominate in outer LA regions.

Our results also confirm a critical role of integrins in the pathobiology of CLL [13, 34–36] further emphasizing that they represent a link between extravasation and survival of leukemia cells. Increased levels of CD49d (α4) expression can be associated with inferior prognosis in IgHV-UM and CD38 expressing cases [37] and the occurrence of trisomy 12 likely associating with the appearance of lymphadenopathies [12–14, 22]. Its expression in CLL cells of LApos patients has been related with enhanced αLβ2 adhesion to ICAM-1 during transendothelial migration [34]. Indeed, CLL cells from patients lacking lymphadenopathy show a defective αLβ2 dependent motility and TEM which is in part corrected through expression of α4β1 in those suffering this organomegaly [34, 38]. This likely explains the lack of LAneg samples to receive prosurvival signal in TEM or when cultured onto ICAM-1 or VCAM-1 and further emphasizes the survival advantage of LApos cells. In agreement with previous studies highlighting a critical role of α4 in the endothelial mediated survival of CLL cells [6, 15, 38, 39]] we further demonstrate that this is especially relevant in lymphadenopathy cases. By contrast, the finding of proapoptotic signaling mediated by these and/or other integrins in the CLL cells when cultured onto ECM ligands or that highlighted in TEM assays were completely unexpected. Integrins dual effects on cell survival, interchanging apoptosis or survival outcomes, have been found in other cell types including hematopoietic tumor cells [40–43]. This can be related with potentiated and sustained adhesion [40] or through unligated integrins [41] or their activation state prior to ligation recruiting caspases [41] and/or ERK rather than Akt kinase [42]. Together these mechanisms and/or other unknown ones may contribute to the presently observed apoptotic outcome when adhering to inflamed endothelium through ligands different from ICAM-1 or VCAM-1.

In keeping with such an integrin dependent survival of CLL cells, cell surface expressed ephrinA4 arises as a critical mediator counteracting apoptosis at initial adhesion steps and nonetheless the prosurvival ones in LApos CLL cells during diapedesis. Repulsive signals mediated by the surface expressed isoform, as previously shown by us [16], along with blood flow could likely contribute to detach the CLL cells from vascular vessels preventing adhesion related apoptotic signals. In LApos patients, by contrast, down regulation of membrane expressed ephrinA4 is needed to allow the firm adhesion to endothelium through α4 and αL integrins [16, 18]. Moreover, we show that ephrinA4 is able of inhibiting high affinity conformational states of integrins through cell surface dependent physical sequestration rather than cell internalization. In supporting this mechanism, we also show enhanced adhesion to endothelium of ephrinA4 silenced CLL cells. The knock-down assays further demonstrate that absence of reverse signaling, but not of ephrinA4 itself, is necessary for LApos samples to go on with diapedesis but not for recruiting survival signals via integrins. The inverse association between ephrinA4 and CD49d molecules further highlights the possible existence of common mechanisms regulating their expression on the CLL cells to assure extravasation. Remarkably, in spite of the low number of tris12 and del11q cases included in the present study, most of them showed low ephrinA4 levels on the surface of CLL cells, a finding that could be linked to the increased tendency to develop lymphadenopathies in these patients.

Accordingly, it is on this basis that binding of the soluble ephrinA4 to endothelial EphA2 arises as a critical mechanism to promote TEM [21–23] coupled to survival in LApos cases. The finding of apoptotic CLL cells near HEV lacking ephrinA4 within the lymphadenopathies indirectly supported also the possible occurrence of this process in vivo. This was further confirmed in chimeric mice assays that, although not physiological at all, represent a good experimental protocol to study in vivo lymph node or bone marrow seeding capacity of human CLL cells [12, 44]. Besides we show that soluble ephrinA4 could promote a transcellular TEM route of CLL cells, as concluded from the VE-cadherin and caveolin-1 stainings of TEM assays, rather than through inducing holes in the HUVEC monolayer as observed in other endothelial studies of permeability using ephrinA1 rather than ephrinA4 [20]. Caveolin-1 mediated TC TEM is coupled to ICAM-1 and VCAM-1 transcytosis [26] similarly to what we have observed in the case of ephrinA4 mediated internalization of EphA2 [16]. Moreover, EphA2 can physically interact with caveolin-1 as indeed demonstrated at a biochemical level [45]. Together these data may shed new light on the long lasting question about the significance of a TC versus a PC route of TEM [26, 27, 46].

**Figure 8:**
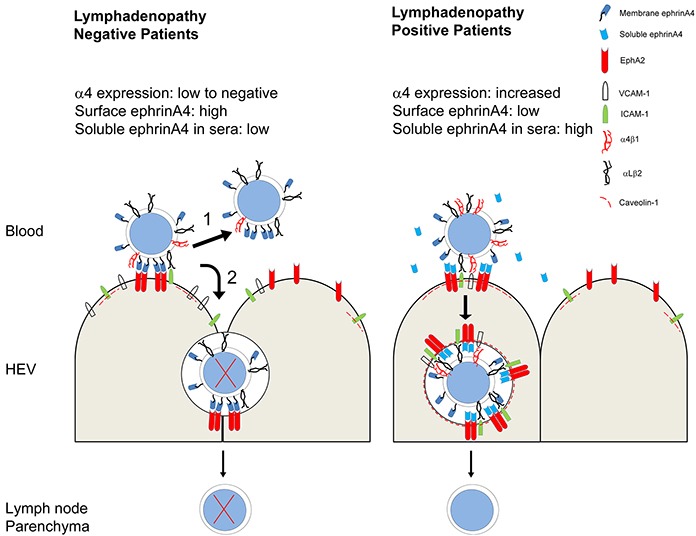
Summary of extravasation decisions and survival outcome of CLL cells according to lymphadenopathy condition determined by integrin and ephrinA4 mechanisms In patients without lymphadenopathy (LAneg, left), CLL cells have low levels of α4 integrin expression impairing their extravasation but not adhesion to endothelium likely through αLβ2. In these conditions, a low frequency of CLL cells extravasate between adjacent endothelial cells (paracellular TEM route) without receiving prosurvival signals and finally die (2). Most CLL cells, however, will detach from endothelium as a result of their high ephrinA4 expression on the cell surface allowing them to continue flowing within blood (1). The levels of soluble ephrinA4 in the sera of LAneg patients are not high enough to counteract the binding of the cell surface expressed one to endothelial EphA2 and detachment. In patients with lymphadenopathy (right) (LApos) expression of α4 integrin is high enough to allow extravasation. In these patients, CLL cell adhesion to endothelium is facilitated by low expression of ephrinA4 on their surface and the high levels of soluble isoform in sera which binds to endothelial EphA2. As a result, adhesion to endothelium will take place facilitating apoptotic signals which, nonetheless, are counteracted in the CLL cells that successfully bind to ICAM-1 and VCAM-1 induced upon EphA2 aggregation. A mechanism coupling EphA2 to caveolin-1 could promote a transcellular TEM route.

Within this context, ephrinA4 could play an instrumental role in ensuring survival of the CLL cells linked to extravasation and lymphadenopathy, as summarized in Figure 8. In conclusion, our results provide new data on the survival mechanisms developed by CLL cells in vivo, demonstrating that extravasation could represent a disadvantageous event to be exploited for preventing nodal dissemination.

## MATERIALS AND METHODS

### Ethics statement

Investigation has been conducted in accordance with the ethical standards and according to the Declaration of Helsinki and national and international guidelines and has been approved by the authors' institutional review board.

### Patients

Patients gave written informed consent before inclusion in this study. Research has been approved by the Ethics and Research Committees of Universidad Complutense de Madrid and H. Morales Meseguer of Murcia according to the principles embodied in the Declaration of Helsinki. The sample size was calculated prospectively for each one of the disease parameters to be studied (Power =0.8; α< 0.05; SPSS Sample Power 3, IBM). Under these criteria a total of 30 subjects were included (Table 1). FISH and IgHV gene mutational status analysis were performed according to previously published methods (Supplementary Material and Methods).

Blood samples were centrifuged onto density-gradient (Histopaque 1.077, Sigma-Aldrich) incubated with lineage specific antibodies (Abs) in PBS (mouse anti-CD3, -CD2, -CD56, -CD14 and -CD13 antigens; BD Biosciences, Europe, Spain) followed by MACS-conjugated anti-mouse secondary Abs (Miltenyi Biotech, Spain) before depletion in an AutoMACS separator (Miltenyi Biotech, Spain). CLL or B cells purities were ≥98%.

Fresh small fragments of lymphadenopathies were available from three patients (CLL patients n. 3, 18 and 28; Table 1) that were embedded in cryoprotective medium (TissueTeck, Leica) and snap-frozen in liquid N2.

### Transendothelial migration (TEM) assays

TEM assays were performed as previously described [16]. Briefly confluent monolayers of HUVECs (PromoCell, Spain) were grown onto the filters of transwell plates (96xwell transwell plates, 5 μm pore size, Corning) and stimulated or not with TNF-α (10 ng/mL; PeProtech, Europe) for 4 hours. HUVEC were left by 6 hours in fresh medium without cytokine before addition of CLL or B cells onto them, for TEM assays, or in the bottom chambers for control suspension cultures (5×10^5^/well). At the indicated time-points, cells were harvested in PBS from the bottom and upper chambers (transmigrated (TM) or non-transmigrated (non-TM) cells in TEM assays, respectively). Where indicated, cellular density was increased in the bottom chambers through direct addition of CFSE fluorescently prestained CLL cells (5×10^5^/well) (5-(and-6)-Carboxyfluorescein Diacetate, Succinimidyl Ester of fluorescein; 10 μM; Thermofisher).

Where indicated, HUVEC monolayers or CLL suspensions were preincubated for 30 min with Fc fragments of human IgG (hFc; 1 μg/mL; Jackson) in the presence or not of saturating amounts of hFc tagged recombinant extracellular domain dimers of human ephrinA4 (ephrinA4Fc; R&D) (0.5 μg/mL; 30 min), EphA2 (EphA2Fc, 0.5 μg/10^6^ cells), VCAM-1 (rhVCAM-1) (0.2 μg/10^6^ cells) or ICAM-1 (rhICAM-1) (all from R&D). Saturating concentrations were determined by flow cytometry using an anti hFc or poly-His antibody as previously described [16].

### Suspension cultures onto plate immobilized integrin ligands

Recombinant cell adhesion molecules ICAM-1 and VCAM-1 (5 μg/mL each) (R&D), extracellular matrix proteins (EMP) including fibronectin (FN, 10 μg/mL), vitronectin (VN, 10 μg/mL), laminin (LM, 10 μg/mL), type I collagen (10 μg/mL) (all from BD) or control protein (BSA; 5 μg/mL; Sigma) were bound to the flat-surface of 96-well culture plates (96 × multiwell culture plates, Corning) for 2 hours at 37°C. Where indicated CLL cell suspensions were incubated for 30 min with recombinant EphA2Fc (0.5 μg/10^6^ cells) and purified hFc fragments of human Igs (1 μg/10^6^ cells, Jackson) and extensively washed in culture medium before addition to culture wells (5×10^5^/well). All cultures were done in 200 μl final volume of freshly prepared RPMI-1640 supplemented with pyruvate (1mM), L-Gln (1 mM) and 1 % FCS in a humidified incubator (5% CO2, 37°C).

### Coculture assays

Confluent monolayers of HUVEC were grown in multiwell plates (96x wells plastic culture plates, Corning). HUVEC activation was performed by providing 10ng/ml TNFα (PeProtech, Europe) to the culture medium for 4 hours followed by extensive washing in fresh medium without cytokine. TNFα activated or untreated HUVEC monolayers were used alive or fixed in 4% paraformaldehyde (PF) in PBS for 20 min followed by extensive washing in fresh medium. CLL cell suspensions (5×10^5^ / well) were harvested from cocultures or control suspension cultures without HUVEC at the indicated time points by extensive washing in PBS containing 5 mM EDTA.

### siRNA knock-down assays

Mixed siRNAs duplexes targeting different exons of *ephrinA4* (Supplementary Material and Methods) or negative control duplexes (Stealth RNAi negative control duplexes, medium-GC, Invitrogen) were nucleofected (300 nM) following manufacturer's recommendations (Amaxa, nucleofection reagents #4DV4XP-3024; 4D-Nucleofector X-unit). EphrinA4 protein knock-down and CLL viability were analyzed by flow cytometry 48 hours postnucleofection.

### Flow cytometry analysis

Cell suspensions were incubated with PE conjugated Annexin-V in HEPES buffer (ImmunoStep, Spain) followed by incubation with 7-AAD solution (5 μg/mL) until analysis in a four-color flow cytometer (FACScalibur, BD; Flow Cytometry and Fluorescence Microscopy Centre, UCM). Absolute cell counts were measured by flow cytometry. Briefly, total recovered cells were suspended in equivalent final volumes of PBS to which equivalent concentrations of fluorescent counting beads were added (CountBrigth absolute counting beads, ThermoFisher). Acquisition was performed at low speed for 1 min. Absolute cell counts were determined according to the following formula:

(Number of B-cell events / Number of bead events) × number of beads added

For immunofluorescent staining cell suspensions were incubated in cold PBS [0.1% bovine serum albumin (BSA)] (2×10^5^ cells/50 μL) with saturating amounts of antibodies to human antigens including: anti-CD19 (FITC, APC or PE), -CD5 (PECy5); FITC or PE-Cy5 anti-CD11a (αL;), -CD29 (β1), -CD18 (β2) or -CD49d (α4)(all from ImmunoStep, Spain); PE conjugated anti ZAP-70 or APC-CD38 (BD). Biotinilated goat-anti human ephrinA4 polyclonal Ab (R&D, Vitro, Spain) in the presence of purified goat IgG immunoglobulins (Jackson Immuno-Research, Europe) followed by streptavidin (SAV)-AlexaFluor-488 (Invitrogen).

### Quantification of soluble ephrinA4 in serum by ELISA

Indirect ELISAs were carried out as previously described [18]. Briefly, plates (MaxiSorp Nunc-Immunoplates, Nunc) were preincubated with an anti-human ephrinA4 goat polyclonal antiserum (R&D) for antigen capture followed by addition of 100 μL serum samples diluted two to eightfold in binding buffer (TBS, 0.5% Tween 20). After 4h incubation, the bound ephrinA4 was detected by incubating wells with a biotinylated anti-ephrinA4 antibody followed by SAV-HRPO conjugate (Jackson-Immunoresearch). Absorbance readings were at 405 nm (reference wavelength 492 nm) on a microplate reader (Bio-Tek Instruments). Standard curves were generated with serial dilutions of a recombinant human ephrinA4 (R&D) (ng/ml).

### Integrin activation state and ligand binding assays

CLL cell suspensions (10^6^ /mL) were preincubated for 30 min (37°C) in RPMI/2%FCS culture medium, with or without MnCl_2_ (1mM), containing purified Fc fragments of human IgG (Jackson). Next, cells were maintained in the same binding medium and incubated 30 min with recombinant human EphA2 (0.5 μg/10^6^ cells). To detect activated VLA4, cells were incubated in cold PBS with PE-conjugated HUTS-21 mAb (Becton Dickinson). To analyze soluble ligand binding, VCAM-1-Fc were preclustered with a PE-conjugated affinity pure F(ab')_2_ fragment goat anti-human IgG, Fc gamma fragment specific (Jackson Immunoresearch) before addition to the EphA2Fcc-preincubated CLL cell suspensions.

### Fluorescence microscopy studies

Fluorescence microscopy studies were performed, accordingly, onto 1) paraformaldehyde fixed (4% in PBS, 30 min) transwell filters from TEM assays, 2) acetone fixed (10 min) tissue cryo-sections from CLL lymphadenopathies (7 μm thick; Leica cryo-cutter,−22°C), 3) CLL cell suspensions adhered onto microscope slides and fixed in paraformaldehyde solution (4% in PBS, 30 min) or 4) CLL-HUVEC co-cultures in 16xwell glass chamber slides (Corning) as previously described [16]. FITC coupled TUNEL detection kit was used according to manufacturer recommendations (Roche). Immunofluorescence stainings were done in humidified chambers in 100 μl PBS (0.1%BSA) containing 0.1 μg/mL antibodies (Supplementary Material and Methods). Nuclei were counterstained with Hoechst (5 μg/mL, 10 min; Thermofisher). Samples were mounted with a non-fluorescent anti-fading mounting solution (ProlongGold, Thermofisher). Confocal images were acquired in a laser confocal microscope system (Leica, TCS SP2 AOBS; Flow Cytometry and Fluorescence Microscopy Centre, UCM).

Image analyses tools and procedures were performed with Image J (Supplementary Material and Methods).

### Xenograft assays in mice

Germ-free BALB/c mice (Charles River Laboratories) were intravenously injected through the tail vein with CFSE prestained (10 μM, Thermofisher) human CLL cells (20×10^6^ cells per mice) in 20 μl sterile PBS solution with or without different concentrations of ephrinA4 purified from serum of CLL patients (Supplementary Material and Methods). Animals were sacrificed by anesthesia 48 hours later and cell suspensions from surgically removed popliteal lymph nodes were stained with an APC conjugated anti mouse CD45 Ab (BD), Annexin-PE and 7AAD for flow cytometry analysis (10^7^ total cells per staining). Human CLL cells were gated according to positivity for CFSE within the mouse CD45 negative cell population (Supplementary Figure S1). The minimum number of animals per experimental condition and CLL sample was prospectively calculated (SPSS Sample Power 3; power, 0.8; α < 0.05). Animal studies were approved by the Ethical and Research Committee in Animal Experimentation of the Complutense University of Madrid.

### Statistical analysis

All in vitro assays were done in triplicate wells and results are shown as mean (±SD). Statistical analyses were performed in SPSS IBM or StatGraphics Centurion XVI. Spearman correlation coefficient was used to measure interrelatedness of variables and significance determined by using the Kruskal Wallis test. Normal distribution of data was determined by Shapiro-Wilks test. Normally distributed data were compared by paired or unpaired, accordingly, two-tailed Student's *t*-test [*, P< 0.05; **, P<0.01; ***, P<0.001; ns, not statistically significant (P≥0.05)].

## SUPPLEMENTARY MATERIALS FIGURES


