# Supplementary material for: EphrinA4 plays a critical role in α4 and αL mediated survival of human CLL cells during extravasation
Source: Oncotarget. 2016 Jun 27;7(30):48481–500. doi: 10.18632/oncotarget.10311 (PMC5217033; doi:10.18632/oncotarget.10311)
Supplement: Supplementary file 1 [file oncotarget-07-48481-s001.pdf]

EphrinA4 plays a critical role in α4 and αL mediated survival of human CLL cells during extravasation

SUPPLEMENTARY MATERIAL AND METHODS

IgHV genes mutation status analysis

The mutational status of IgHV genes was analyzed according to ERIC recommendations by using VH family-specific forward primers in combination with one consensus reverse primer to the JH region in independent PCR reactions (BIOMED-2 protocol;[17]). Cleaned up PCR products were directly sequenced and forward and reverse sequences aligned to IMGT immunoglobulin database (IMGT/VQUEST tool; International ImMunoGeneTics information system, http://imgt.cines.fr)[18,19]. A germline homology of ≤ 98% was considered as unmutated (UM).

Interphase Fluorescence in situ hybridization (FISH) was carried out to detect cytogenetic abnormalities at 13q, 12, 11q and 17p arms by using Vysis (Abbott) probes 17p13.1, 11q22.3, 13q14.3 and 13q34 according to manufacturer's guidelines. A cut-off of 5% was used for all abnormalities with the exception of 17p-deletion in which a cut-off of 10% was applied. In each case, at least 200 nuclei were evaluated.

EphrinA4 siRNA sequences

Stealth Select siRNAs duplexes (Invitrogen, Thermofisher, Spain); Stealth EFNA4 Primer pair 1: (SENSE STRAND) CCC UGC CCU UUG GCC AUG UUC AAU U; (AS STRAND) AAU UGA ACA UGG CCA AAG GGC AGG G; Stealth EFNA4 PP-2: (S. ST.) CCA GAG AGU UCU GGC CAG UGC UUG A; (AS. ST.) UCA AGC ACU GGC CAG AAC UCU CUG G; Stealth EFNA4 PP-3: (S.ST.) GGU GCC AAU UCA GAC CGA CAA GAU G; (AS. ST.) CAU CUU GUC GGU CUG AAU UGG CAC C).

EPHA2 siRNA (VALIDATED stealth, invitrogen))

S.ST. GCA AGG AAG UGG UAC UGC UGG ACU U
AS. ST. AAG UCC AGC AGU ACC ACU UCC UUG C

EphA2 siRNA knock-down in HUVEC

Validated RNAi oligo duplexes (EphA2 Validated Stealth RNAi, Invitrogen, LifeTechnologies) were transfected to HUVECs either adhered to culture plates (Lipofectamine RNAiMAX, Invitrogen) or in suspension (Nucleofection technology; Amaxa 4D-Nucleofector for HUVEC; Cat. No. V4XP-5024; Lonza) following manufacturers recommendations before and after TNF-α treatment. Assessment and optimization of RNAi delivery into HUVEC within each approach was performed by transfecting AlexaFluor conjugated oligos (BLOCK-iT AlexaFluor Red Fluorescent Oligo; Invitrogen; or AlexaFluor488 ALLSatrs Negative Control, Quiagen) for fluorescence microscopy or flow cytometry determination of transfection efficiency. Negative control RNAi oligos (Stealth RNAi negative Control Duplexes, Medium GC; Invitrogen) transfection experiments were done as a control for sequence independent effects following RNAi delivery. EphA2 knockdown was determined at 24 to 48 hours post-transfection by both flow cytometry or fluorescence microscopy analyses with either PE-conjugated anti human EphA2 (Santa Cruz Biotechnology) or unconjugated Ab followed by secondary Alexa Fluor donkey anti goat (LifeTechnologies).

Following transfection, HUVEC were allowed to grow in optimal conditions for the establishment of 100% confluent monolayers.

Confocal microscopy reagents and image analyses procedures

Antibodies against human antigens were: anti-CD31 (AlexaFluor (AF) 647 conjugated, raised in mouse; Biolegend), -PNAd (biotinilated, rat; Santa Cruz), -ephrinA4 (biotin coupled, goat, R&D), -EphA2 (unconjugated, rabbit, SantaCruz), -ICAM-1 (AF488, mouse; Biolegend), -caveolin-1 (unconjugated, rabbit; SantaCruz), -VE-Cadherin (unconjugated, mouse; Santa Cruz); -α4 or -αL integrin chains (AF647 conjugated, Biolegend). Secondary antibodies were anti-goat AF488, anti-rabbit AF594 or anti-mouse AF543 (donkey; Thermofisher); AF488 or AF543 conjugated Streptavidin (Thermofisher); biotin conjugated anti poly-His (R&D).

Transwell filters were scanned throughout at 1 µm z-step resolution (4 scanned fields per filter; 20x oil immersion objective). Total nuclei (Hoechst staining) and apoptotic ones (FITC-TUNEL staining) were discriminated and counted according to color intensity threshold and particles size ("3D Objects Counter" tool, Image J 1.49s, 64 bits).

Apoptotic cells counting in CLL lymphadenopathies (>3 microscopy fields per section; >10 sections per sample, ≤200 µm between sections; 20x oil immersion objective) were based in intensity and size thresholds ("Analyze Particles" tool, ImageJ). Distance measurements (µm) of apoptotic nuclei to PNAd stained vascular vessels were calculated on calibrated images by means of manual line tracing (ImageJ). EphrinA4 content within EphA2 vascular

vessels was measured after tracing regions of interest (ROIs) around EphA2 stained vessels and integrated intensities of ephrinA4 staining (Measure, ImageJ).

Transcellular (TC) or paracellular (PC) transmigrating CLL cells were discriminated and counted (ImageJ) according to their presence within caveolin-1 holes at > 1 µm of interendothelial junctions or at VE-Cadherin interendothelial junctions, respectively. Nuclei not fulfilling either criterion were referred to as unclassified. Only nuclei inside of HUVEC, as determined by Z-side views of stacks, were considered as crossing the HUVEC monolayer.

Purification of human ephrinA4 from CLL patients' sera

Affinity columns were prepared with goat anti-human ephrinA4 antibody (R&D) cross-linked to protein G-agarose (Sigma) through dimethyl pimelimidate dihydrochloride (DMP, SIGMA). Ten mL of three CLL patient' sera were dialyzed in cold PBS for 12 hours and then applied to affinity columns. Bound ephrinA4 was eluted in 100mM glycine buffer (pH 2.5) followed by 12 hours dialysis in cold PBS for further use.

SUPPLEMENTARY FIGURES

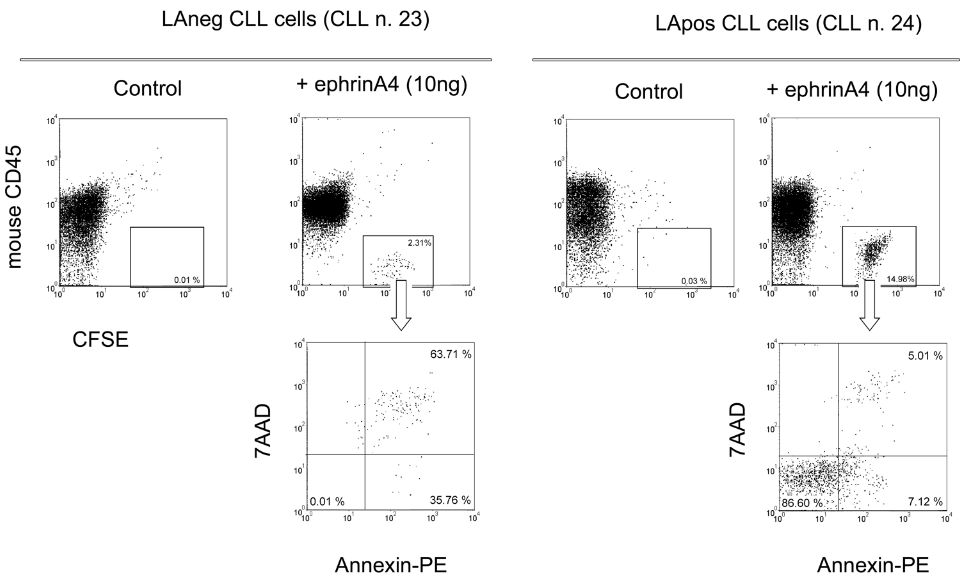

Supplementary Figure S1: Flow cytometry analysis of human CLL cells in popliteal lymph nodes of mice. Germ-free Balb/c mice were intravenously inoculated through the tail vein with CFSE pre-stained (5μM, 1 h; 2×10^7 cells / mice) CLL cells from patients having or not lymphadenopathy (LApos and LAneg, respectively) in 100 μL sterile PBS containing or not (control) different concentrations of a human ephrinA4 purified from serum of patients. Mice were sacrificed 24 four hours later and cell suspensions from surgically removed popliteal lymph nodes were stained with an APC conjugated antibody against mouse CD45 antigen (mCD45; BD), PE conjugated Annexin-V and 7AAD followed by flow cytometry analysis. Human CLL cells were identified according to CFSE staining within the mCD45 negative population (upper plots, rectangles). Annexin-V-PE/7AAD staining was analysed within the gated $CFSE^+$ $mCD45^{neg}$ population (lower plots).

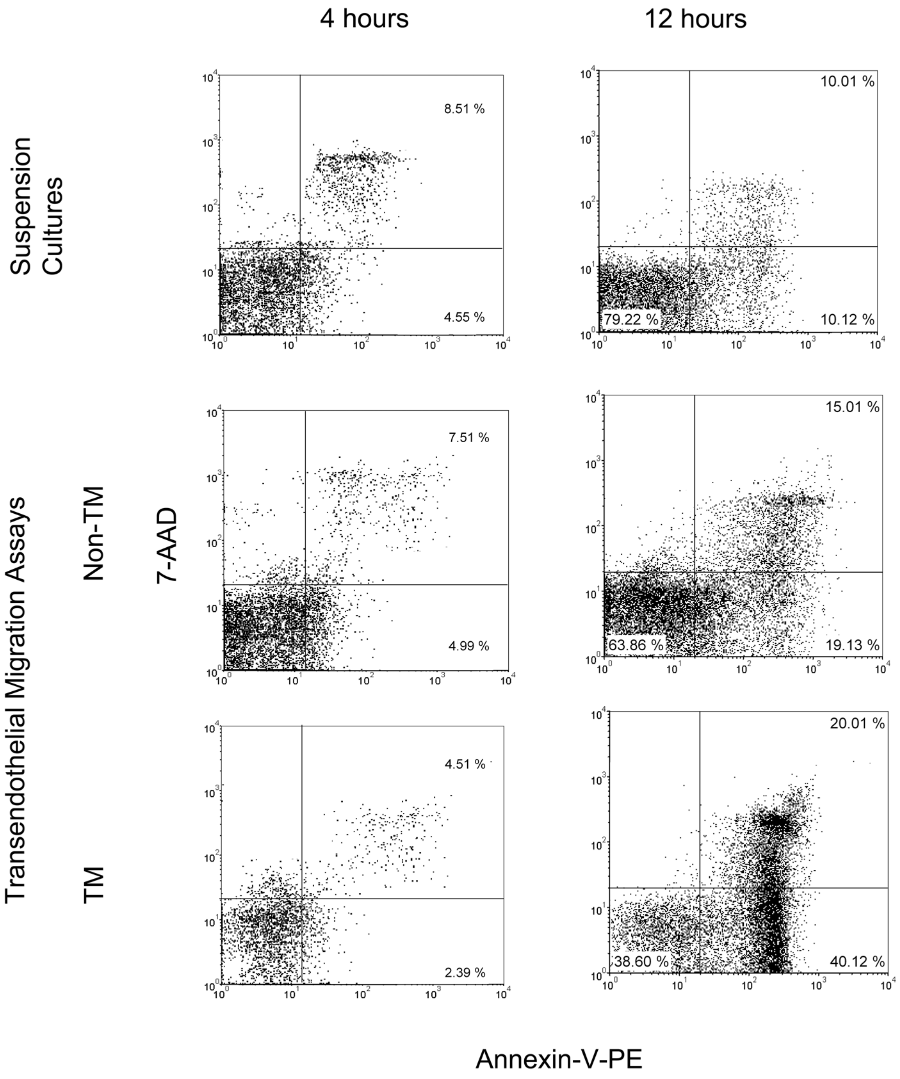

Supplementary Figure S2: Flow cytometry analysis of apoptosis and cell viability either in suspension cultures or transendothelial migration assays. CLL cells were cultured either in suspension alone or kept transmigrating through TNFα treated confluent HUVEC monolayers (5×10^5 cells per well). Cells were harvested at the indicated time points and double stained with Annexin-V-PE/7AAD for flow cytometry analysis. Percentage of viable (lower left quadrants, AnnexinV^{neg} 7AAD^{neg}) and early apoptotic cells (lower right quadrants; AnnexinV^{pos} 7AAD^{neg}) were analyzed in the suspension cultures and in the upper and bottom chambers of transwells (non-transmigrated (Non-TM) and transmigrated (TM) cells, respectively). The most significant changes in apoptotic and viable cells were detected after 12 hours in the ten samples analysed. A representative sample out of 10 is shown.

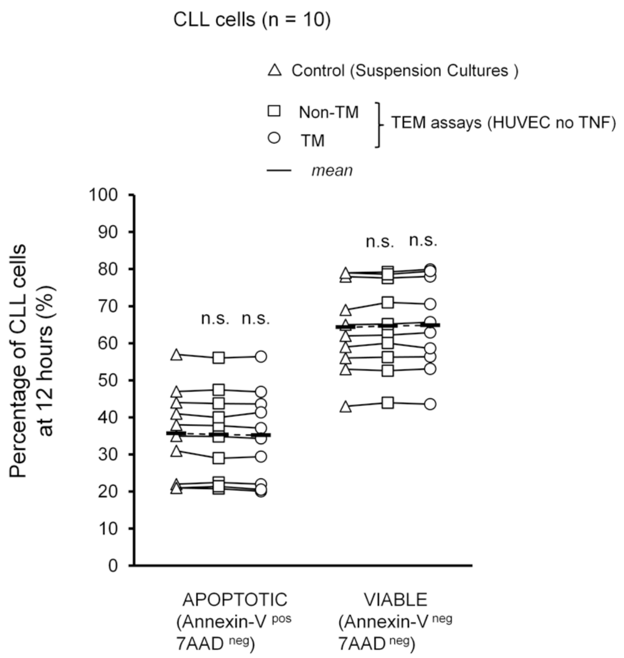

Supplementary Figure S3: TEM through unstimulated HUVEC monolayers does not alter viability of CLL cells. CLL cells from ten patients were cultured in suspension or kept transmigrating through unstimulated confluent HUVEC monolayers (5×10^5 cells per well). Cells were harvested at 12 hours and double stained with Annexin-V-PE/7AAD for flow cytometry analysis. Non-TM, non-transmigrated; TM, transmigrated. Statistical significances were determined by two-tailed Student's t-test (* < 0.05; ** <0.01; *** <0.001).

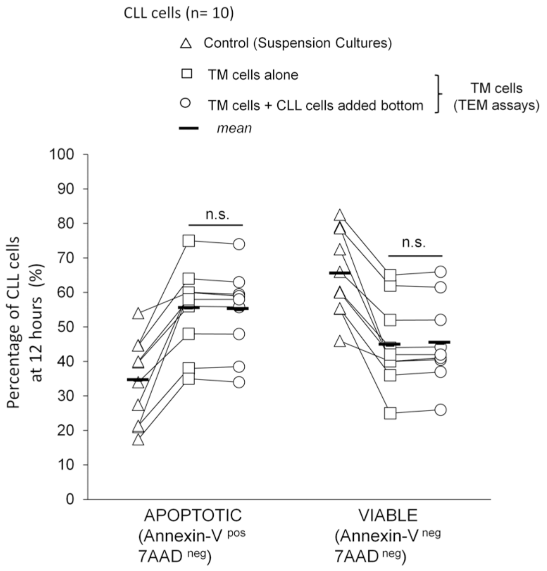

Supplementary Figure S4: Cellular density in bottom chambers does not explain the drop in viability of TM CLL cells in TEM assays. CLL cells (5×10^5 per well) (patients n. 1-10) were maintained in culture alone without HUVECs (suspension cultures) or transmigrating through TNFα treated HUVEC monolayers in transwell plates (TEM assays). The effect of cellular density in the viability of TM cells was analyzed by adding 5×10^5 CLL cells directly into the bottom chamber at the initiation of TEM. The added CLL cells were prestained with CFSE (5 µM, 10^7/mL, 1hour) to distinguish them from TM cells (CFSE negative) by flow cytometry. Percentages of viable and apoptotic cells were measured by flow cytometry analysis of Annexin-V-PE/ 7AAD double staining. The increased cellular density in bottom chambers did not significantly change the percentage of apoptotic and viable cells found in the TM fraction. Statistical significances were determined by two-tailed Student's t-test (* < 0.05; ** <0.01; *** <0.001).

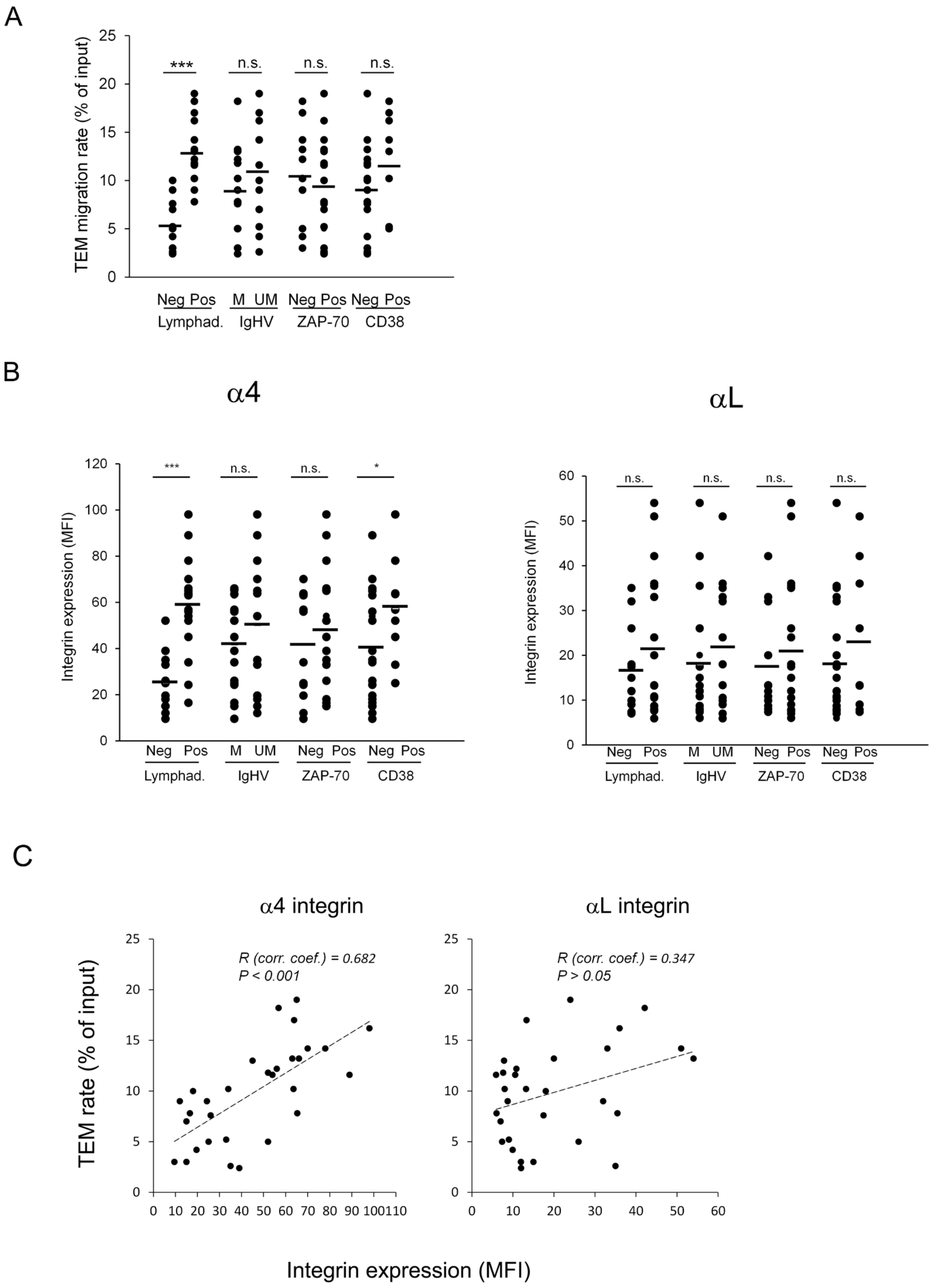

Supplementary Figure S5: Correlation analysis between expression of α4 and αL integrin chains and both disease parameters and TEM rate of samples. A. CLL cells from 30 patients were maintained transmigrating through TNF-HUVEC for 12 hours. The absolute number of transmigrated CLL cells in the bottom chambers were measured by flow cytometry and expressed as percentage of total input cells (TEM rate). **B.** Cell surface expression of the indicated integrin chains was measured by flow cytometry in the CLL cells of 30 patients after staining with antigen specific antibodies and correlated with the indicated disease parameters (B) or TEM rate of samples **C.** Statistical significances were determined by two-tailed Student's t-test (* < 0.05; ** <0.01; *** <0.001). Spearman correlation coefficient, R.

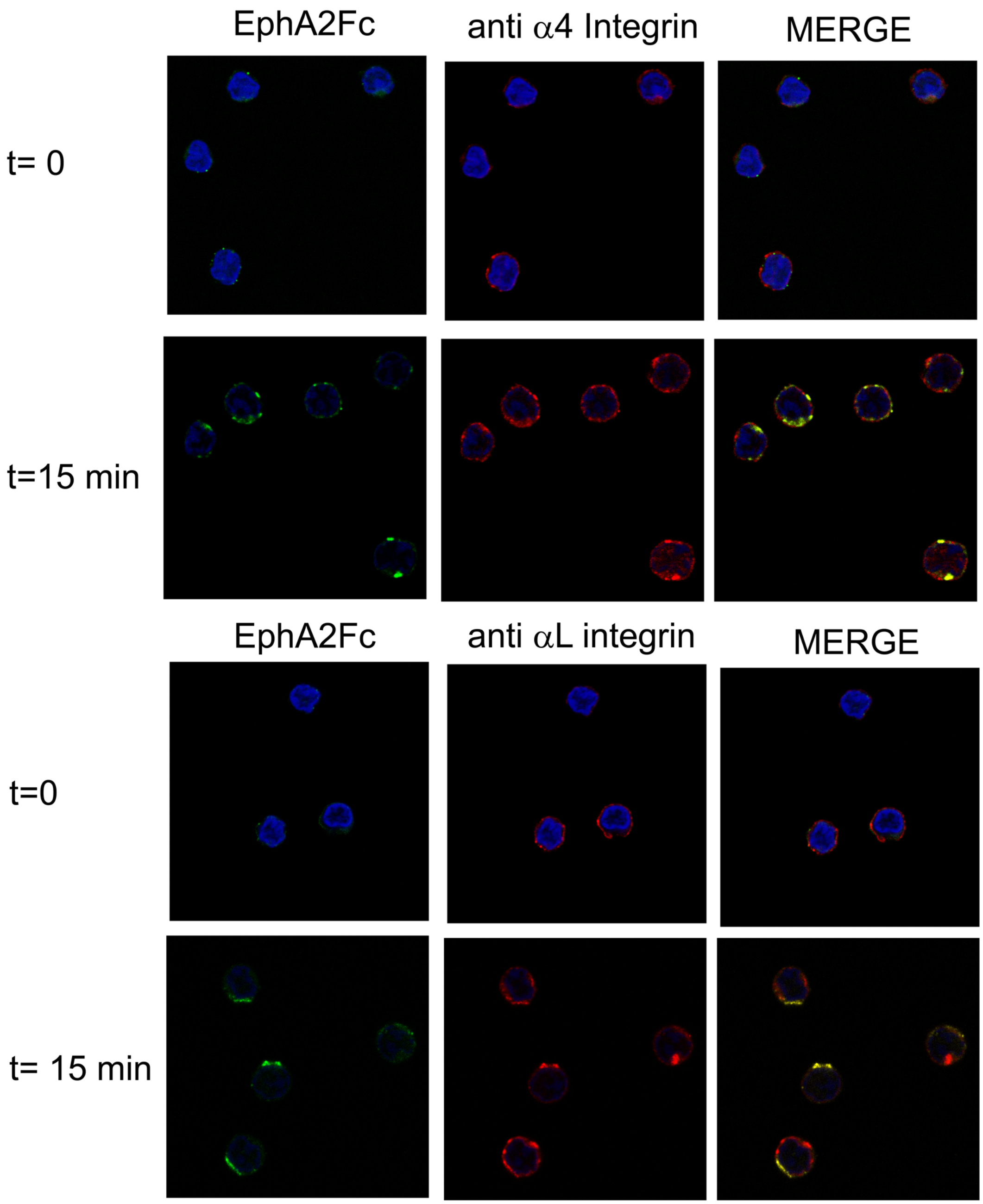

Supplementary Figure S6: Fluorescence confocal microscopy study of ephrinA4 cell surface clustering through recombinant EphA2Fc and integrins sequestration. CLL cell suspensions (5×10^5) were incubated with saturating amounts of poly-His tagged recombinant extracellular domains of human EphA2 (EphA2Fc) for 30 min followed by another 30 min incubation with a biotin conjugated anti-His mAb. After washing, cells were incubated with AlexaFluor 488 conjugated streptoavidin in cold PBS (t=o) or at 37°C in culture medium during adhesion to microscope slides for 15 min to induce ephrinA4 clustering on the cell surface. After fixation in paraformaldehyde (4%), slides were incubated with AlexaFluor647 conjugated antibodies to the indicated integrin chains and nuclei counterstained with Hoechst. A representative experiment is shown.
